# Structure-Related Gelling of Pectins and Linking with Other Natural Compounds: A Review

**DOI:** 10.3390/polym10070762

**Published:** 2018-07-11

**Authors:** Diana Gawkowska, Justyna Cybulska, Artur Zdunek

**Affiliations:** Institute of Agrophysics, Polish Academy of Sciences, Doświadczalna 4, 20-290 Lublin, Poland; d.ganczarenko@ipan.lublin.pl (D.G.); a.zdunek@ipan.lublin.pl (A.Z.)

**Keywords:** food chemistry, fruits and vegetables, pectin, gelation, interaction

## Abstract

Pectins are polysaccharides present commonly in dicotyledonous and non-grass monocotyledonous plants. Depending on the source, pectins may vary in molecular size, degrees of acetylation and methylation and contents of galacturonic acid and neutral sugar residues. Therefore, pectins demonstrate versatile gelling properties and are capable of forming complexes with other natural compounds, and as a result, they are useful for designing food products. This review focuses on the structure-related mechanisms of pectin gelling and linking with other natural compounds such as cellulose, hemicellulose, ferulic acid, proteins, starch, and chitosan. For each system, optimal conditions for obtaining useful functionality for food design are described. This review strongly recommends that pectins, as a natural biocomponent, should be the focus for both the food industry and the bioeconomy since pectins are abundant in fruits and may also be extracted from cell walls in a similar way to cellulose and hemicellulose. However, due to the complexity of the pectin family and the dynamic structural changes during plant organ development, a more intensive study of their structure-related properties is necessary. Fractioning using different solvents at well-defined development stages and an in-depth study of the molecular structure and properties within each fraction and stage, is one possible way to proceed with the investigation.

## 1. Introduction

Pectins belong to the family of polysaccharides present in the primary cell walls and in the middle lamella of higher plants. The largest amounts of pectin are found in dicotyledonous and non-grass monocotyledonous plants [1]. Pectin biosynthesis takes place in the Golgi vesicles [2]. Released pectins are characterized by a high degree of methylation, which may be modified by the activity of pectin methylesterases. Some regions of pectin are involved in the formation of calcium ions-pectin cross-links. These polysaccharides are very important for the growth and development of plants due to their impact on both the rigidity and integrity of plant tissues [3,4]. They play a role in ion transport, define the porosity of cell walls, and have an influence on the activation of the immune system of plants [5,6]. The numerous functions of the various pectins depend on their structure and concentration in cell walls, and that is highly variable due to the chemical and enzymatic modifications which occur during the growth and ripening periods, storage, and processing of fruits [4,7]. Pectins are one of the most commonly used plant polysaccharides for food design due to their versatile gelling properties which are utilized in jellies, jams, marmalades, fruit juice, confectionery products, etc. [8,9]. Pectins are also pro-health as essential constituents of soluble dietary fibre [10]. It has been demonstrated that pectins contribute to lowering the cholesterol level in blood [11], bind lead and mercury in the gastrointestinal tract [12], and have an anti-cancer role [13].

Due to very diverse and variable pectic polysaccharides, it is commonly accepted that the functionality of pectins for food design depends on their structure and properties. However, on the other hand, due to the variability of plant pectin sources, controlling the pectin structure extracted from plants for the particular purpose of various experiments is extremely difficult. Therefore, the comparison of results of different experiments on different plants and different maturity stage is obviously often ambiguous. In this review, we took on the challenge to present the gelling properties of pectins and their ability to link with other natural components that might be used in food products.

## 2. Structure of Pectins

### 2.1. The Pectin Family

Pectins are composed mainly of an α-1,4-d-galacturonic acid residue. Different pectin domains may be distinguished: homogalacturonan (HG), rhamnogalacturonan I (RGI), rhamnogalacturonan II (RGII), xylogalacturonan (XGA), apiogalacturonan (AGA), arabinan, galactan, arabinogalactan I (AGI) and arabinogalactan II (AGII).

**Figure 1:**
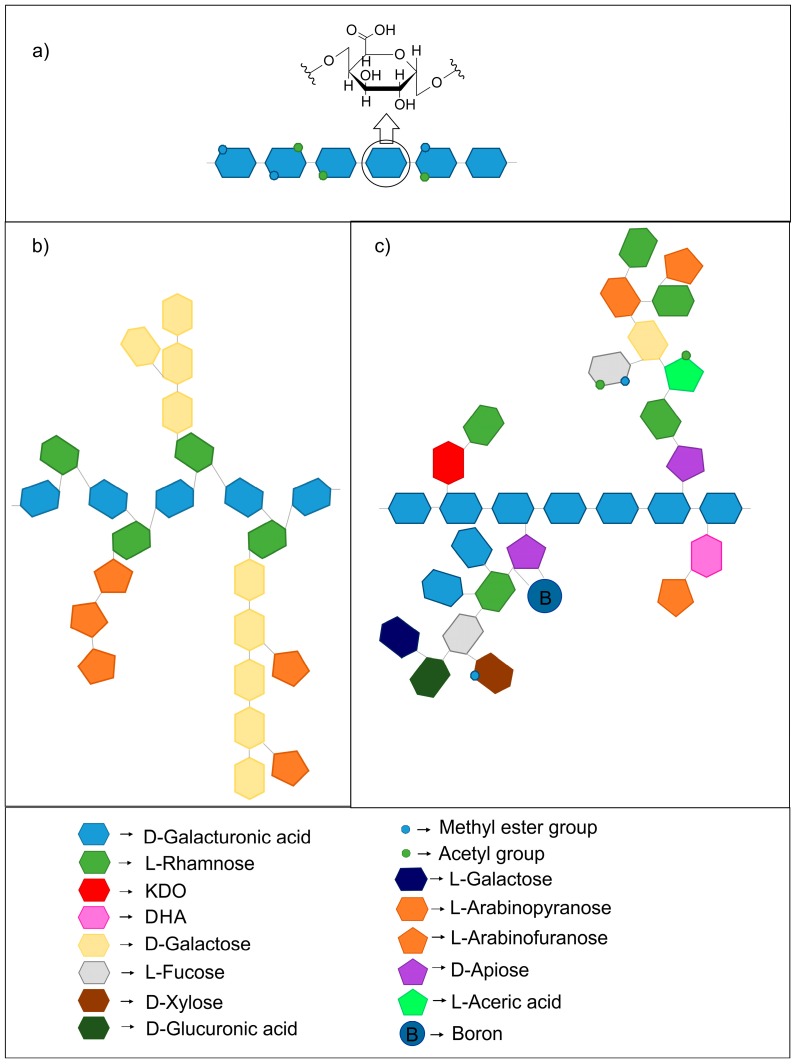
Schematic structure of pectin polysaccharides: (**a**) homogalacturonan; (**b**) rhamnogalacturonan I; (**c**) rhamnogalacturonan II, based on: [1,36,37].

The most abundant domain in the pectic macromolecule is homogalacturonan (Figure 1a) which accounts for about 60% of all pectins in cell walls [4]. The HG molecule consists of a linear chain of α-1,4-d-galacturonic acid units in which some of the carboxyl groups are esterified with methanol or/and acetyl groups are present at O-2 or/and O-3. In the cases of apple, citrus, and sugar beet the backbone of HG is composed of at least 72–100 d-galacturonic acid residues [14]. The degree of methylation (DM), expressed as the ratio of methyl-esterified carboxyl groups to the total amount of galacturonic acid units, is one of the key parameters related to gelling capability and may be determined using infrared spectroscopy [15,16]. DM is calculated based on the areas under the peaks at 1600–1630 cm^−1^ and 1740 cm^−1^ associated with the antisymmetric stretching vibrations of COO^−^ and the C=O stretching vibration of the ester. The technological classification of pectin is based on the degree of methylation, high-methoxy (HM) pectins have over 50% of their carboxyl groups esterified with methanol, low-methoxy (LM) pectins between 5% and 50%, and pectic acid below 5% [17,18].

Rhamnogalacturonan I consists of repeating residues of α-1,4-d-galacturonic acid and α-1,2-l-rhamnose (Figure 1b). The length of the backbone of RGI isolated from suspension-cultured sycamore cells is about 100–300 repeating units [19]. However, RGI may be much shorter consisting about 20 residues of this disaccharide, this has been reported for sugar beet [20]. At C-4 of the rhamnose residues, side chains containing galactose and/or arabinose residues are attached. The side chains are composed of a single sugar residue or combined chains of arabinans, galactans or arabinogalactans [19,21]. The proportion of branched rhamnose units is 20–80% and depends on the origin of the polysaccharides [7]. It is assumed that HG and RGI are covalently linked [22]. These polysaccharides cannot be separated without a chain-cleavage using for example, enzymes such as endopolygalacturonase [23] or chromatographic fractionation methods [24,25].

Rhamnogalacturonan II is composed of a backbone that consists of 7–9 galacturonic acid residues branched with 4 side chains (at C-2 and C-3) which may include arabinose, apiose, fucose, galactose, rhamnose, aceric acid, glucuronic acid, galacturonic acid, 2-*O*-methyl-xylose, 2-*O*-methyl-fucose, 3-deoxy-lyxo-2-heptulosaric acid (DHA), and 3-deoxy-manno-2-octulosonic acid (KDO) [26], (Figure 1c). RGII isolated from pea stems and suspension-cultured sycamore cells is mainly covalently linked with boron [27]. It may have an influence on the formation of the complex structure of the cell wall, which contains pectin polysaccharides such as HG, RGI and RGII. In vitro studies indicated the formation of dimers RGII via apiosyl residues.

Xylogalacturonan is a polymer containing a homogalacturonan backbone with xylose residues attached to O-3 of some galacturonic acid units [28]. The number of branches and the degree of methylation of HG may vary considerably depending on the source of the polysaccharides. In the apiogalacturonan macromolecule, one or two apiosyl residues connected with each other are linked to galacturonic acid units [29]. Arabinans consist of chains of α-1,5-l-arabinofuranosyl units with attached α-arabinofuranosyl residues at C-2 or/and C-3 in the arabinose molecule [30]. Most of the arabinans present in primary cell walls are side chains of rhamnogalacturonan I [31].

Galactans composed of β-d-galactose units linked by β-1,4-glycosidic bonds are the most common type of side chain of rhamnogalacturonan I in potato [32].

Arabinogalactan I is composed of a backbone of β-1,4-d-galactopyranosyl residues with attached short chains of α-1,5-arabinose residues at C-3 in the galactose molecule; however, the binding of β-galactose to the galactan chain through the O-6 position may also occur [7,33,34]. Arabinogalactan II similarly contains chains of β-d-galactopyranosyl units, but glycosidic linkages occur at C-1, C-3, and C-6 in galactose molecules [35]. This type of arabinogalactan is highly ramified [7]. It may also include an l-arabinopyranosyl residue on the end of the chain of β-1,6-d-galactopyranosyl units. Longer chains of α-1,3-arabinose residues may be attached to the backbone of β-1,3-d-galactopyranosyl units. Furthermore, arabinofuranosyl residues or arabinopyranosyl residues may be joined at C-6 in galactose molecules of this backbone. Arabinogalactans I and II may be side chains of rhamnogalacturonan I [31].

### 2.2. Pectin Sources

The plant sources used for pectin extraction together with their content of galacturonic acid and reported yield are summarized in Table 1. On a commercial scale, pectins are prepared from apple pomace and citrus peel by acid extraction, filtration, and precipitation by alcohol. The pectin content of apple pomace and citrus peel is about 10–15% and 20–30% of dry matter, respectively [38]. Sunflower head residues and sugar beet have also been used as pectin-rich sources that contain about 10–20% pectin on a dry weight basis [39].

### 2.3. Structure of Pectins Related to Solubility

Pectins present in plant cell walls vary in their solubility [57], in terms of extractability. The extraction of different fractions based on the preparation of alcohol-insoluble residues (AIR) and sequential extraction using water, a chelating agent such as CDTA or EDTA, and sodium carbonate [58,59]. Depending on the solvent used each pectin fraction is identified as a water-soluble pectin (WSP), chelator-soluble pectin (CSP), and diluted alkali-soluble pectin (DASP). The WSP fraction contains molecules, which are non-ionic and non-covalently bound to the cell walls [57]. The CSP fraction involves pectin polymers linked to the cell wall by ionic bonds, for instance via calcium ions. This type of pectin can also be extracted using imidazole [60]. The DASP fraction contains pectins, which are bound to the cell wall by covalent ester bonds [57].

The amount of galacturonic acid in each fraction is usually different, and varies by plant species and, moreover, it changes during the physiological development of the plant. For instance, for carrot, the galacturonic acid content of the WSP, CSP, and DASP fractions obtained from cell walls directly after harvest were about 20, 65, and 95 mg/g AIR [58]. During 5 months postharvest storage at a temperature of 2 °C the content in the CSP fraction increased, in the WSP fraction it increased until the third month of storage and then it decreased whereas in the DASP fraction it changed only slightly during the whole period of storage. As a result, the total galacturonic acid content increased with storage time which may be connected with the formation of new pectins [58,61]. For plum fruit, the galacturonic acid content (characterized as anhydrouronic acid content) in the same fractions was much higher: 523–665, 536–845, and 469–780 mg/g AIR, in the WSP, CSP and DASP fraction respectively [10]. When the galacturonic acid content was evaluated in pectin fractions obtained from plum pomace, the galacturonic acid content was 478–690, 641–690, and 617–858 mg/g AIR, respectively. For blueberry, the WSP, CSP, and DASP fractions contained 516, 627 and 580 mg/g of galacturonic acid in the sample, respectively [59]. A comparison of galacturonic acid content from different experiments on different fruit is more difficult due to the specific solvent used or data presentation. Nunes et al. extracted fresh plum AIR using water, imidazole, and sodium carbonate at 4 and 20 °C, and obtained 48–51, 64–76, 85–87, and 59–61 mol % of uronic acid in these fractions expressed in relation to the total cell wall sugars [62]. Sodium acetate buffer, EDTA, 0.05 M sodium hydroxide, and 6 M sodium hydroxide were used to extract pectin from blackcurrant and bilberry [63]. The galacturonic acid content of blackcurrants was 84, 88, 58 and 6 mol % (of total sugars), respectively, while in the case of bilberries the galacturonic acid content was 83, 83, 70 and 5 mol% (of total sugars), respectively.

As with the galacturonic acid content, the degree of methylation of pectin fractions extracted from different sources can vary and depends mainly on the physiological state of plant. The degree of methylation in the WSP fraction from mature carrot was dependant on the maturity stage and amounted 71.8–78.8% [58]. For the WSP, CSP, and DASP fractions isolated from blueberry powder, the degree of methylation was 36%, 28%, and 26%, respectively [59]. Kosmala et al. extracted the fractions of pectins from plum fruit and pomace, where the degree of methylation of WSP fractions was 43–69% and 53–57%, respectively. In the case of the CSP fraction, this parameter was 24–38% for fruit and 18–39% for pomace [10]. The degree of methylation was 36–51% and 65–75% in water and imidazole extracts obtained from fresh plum AIR [62]. It may be concluded that the type of solvent (used for extraction) may have an influence on the degree of methylation of the pectins.

Numerous studies of the molecular size of pectin were conducted using, among other techniques, viscometry [64], end group analysis [65] or high-performance size exclusion chromatography [66]. High-performance size exclusion chromatography is commonly used to determine pectin molecular weight for the food industry [67]. Depending on the method of extraction and pectin source, the molecular weight may differ over a wide range. Pectin extracted from potatoes by various acids (acetic acid, citric acid, hydrochloric acid, nitric acid and sulphuric acid) had a weight-average molecular weight in the range of 2.3–3.2 × 10^5^ g/mol [68]. Some differences in the values of this parameter resulted from a different tendency to hydrolyse by particular acids. The weight-average molecular weight of sunflower pectin, isolated using ammonium oxalate, was ~6 × 10^5^ g/mol [54]. Pectin fractions extracted from apple pomace by a chelating agent and sodium carbonate had a higher peak molecular weight (defined as the molecular weight of the highest peak), 16 and 24 × 10^5^ g/mol, respectively, than commercial apple pectin, ~6 × 10^5^ g/mol [69]. 

**Figure 2:**
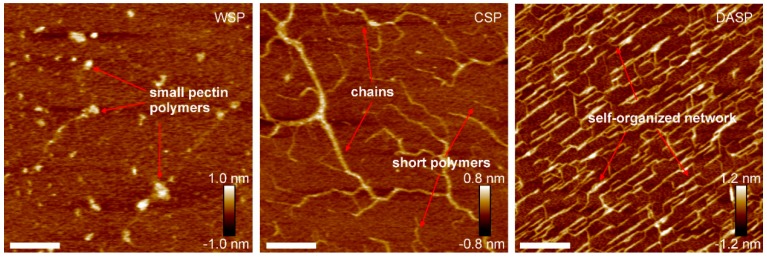
AFM images of water-soluble pectin (WSP), chelator-soluble pectin (CSP), and diluted alkali-soluble pectin (DASP) fractions obtained from apples (cultivar Idared); white bar indicates 200 nm.

Recently, atomic force microscopy (AFM) was successfully used for the characterization of molecular structures of water-soluble, chelator-soluble, and diluted alkali-soluble pectins. The WSP fraction isolated from carrot had a high content of small pectin polymers which had a diameter and maximum height of about 22 nm and 0.2–0.4 nm, respectively [70]. A small number of elongated structures was observed as well. Short pectin polymers in the WSP fraction extracted from pear cell walls were also found in a study by Zdunek et al. [71]. Large polymers and blocks in the WSP fraction were obtained from the cell walls of fresh peaches by Zhang et al. [72]. Cybulska et al. showed that the polymer size in the WSP fraction (extracted from carrots) changed slightly during storage (up to about 24 nm) [70]. In the case of the CSP fraction from carrot roots, a mixture of both chains and short pectin polymers were observed. The skeleton length was more than 400 nm for a substantial part of the pectin chain, the molecules were also highly branched. The average value of the height of the chains was 0.21 nm. It has been suggested that rhamnogalacturonan I is attached to homogalacturonan chains forming the branched structure of the CSP fraction. The evidence for this came from FT-IR spectra representing absorbance bands at 1040, 975, and 945 cm^−1^ associated with the presence of galactose and arabinose residues in rhamnogalacturonan I [73]. Branched structures were also noted in the CSP fraction from pears [71]. After 3 months of storage, the length of the side chains in the CSP fraction (from carrots) was reduced and more linear and shorter chains were observed [70]. In a study by Kirby et al. branched structures were also found in the CSP fraction from unripe tomato cell walls [74]. The contour length of the molecules ranged from 20 to 540 nm. An interesting structure was elucidated for DASP embedded on freshly cleaved mica. The DASP fraction self-organized in a regular network on mica [70]. The height of most of the molecules was 0.8 nm and the average value of the height was 1.2 nm. Straight, long molecules with side branches were observed based on an analysis of the AFM data. The angle between the molecule and its side branch was observed to be 119°. Long molecules also had characteristic bending locations with an angle of 118°. The well-ordered network of DASP fractions was connected with these angles. It has been theorized that arabinose and galactose units occur in this fraction, based on FT-IR data [70]. The DASP fraction, extracted from pear cell walls, also formed a network on mica [71]. Typical AFM images of WSP, CSP, and DASP fractions obtained from apples (cultivar Idared) are shown in Figure 2.

Posé et al. have suggested that the rhamnogalacturonan I structure is present in the DASP fraction due to absorbance bands with high intensities at 1075 and 1047 cm^−1^ [75]. Absorption band maxima at these wave numbers were observed in rhamnogalacturonan I by Kacuráková et al. [76]. During storage the regular network structure of the DASP fraction extracted from carrots was degraded and the proportion of the shortest molecules increased [70]. Linear and branched structures in the CSP and DASP fractions extracted from strawberry fruits were found by Posé et al. based on AFM studies [75]. The level of branching for both fractions was 9%, but in the case of the DASP fraction, a higher content of multi-branched molecules was observed.

It may be concluded that the fractionation of pectin using various solvents allows pectin fractions to be obtained which differ in chemical composition, molecular structure and other properties.

## 3. Gelling Capacity

The source and the method of extraction have an influence on the structure and properties of pectin such as viscosity and gelling ability, and thus their application in the food industry.

Many parameters have an influence on the functional properties of pectins. The solubility of pectins in aqueous solution depends on the degree of methylation, molecular weight, counterions present in the solution, temperature, and pH [77,78]. Solubility is greater when the degree of methylation is higher, however, it decreases when polymer size increases [3]. An increase in temperature in weakly acidic and neutral conditions may cause the degradation of pectin and a change in solubility as a result β-elimination reaction. The mechanism of the β-elimination reaction is related to the cleavage of the glycosidic bond at C-4 and the removal of the hydrogen atom at C-5 of the galacturonic acid unit [79,80]. A double bond is formed as a result of this reaction. An increase in temperature and pH, a high degree of methylation, the presence of monovalent salts and EDTA accelerate the β-elimination reaction [3]. The susceptibility to the β-elimination reaction was higher in the WSP fraction than in the CSP and the DASP fraction isolated from pretreated carrot tissues [81]. It was connected with the higher degree of methylation of the WSP fraction in comparison to other fractions. In food technology the β-elimination reaction occurs during thermal processing of food at pH > 4.5 and causes a loss of texture and a decrease in the viscosity of food products [3].

The viscosity of pectin solutions and gel formation depend on their solubility. Generally, parameters, which cause a decrease in solubility increase viscosity and gelation [38]. The influence of concentration and temperature on the viscosity of pectin solutions extracted from orange peel was investigated by Kar and Arslan [82]. The viscosity increased with the increasing concentration of pectins and decreased when the temperature increased. Increasing the concentration of pectins may reduce intermolecular distances and enhance intermolecular interactions such as hydrogen bonding. At higher temperatures the kinetic energy of molecules increases, thus intermolecular distances also increase and viscosity declines [82]. Three phases in the increase of viscosity due to pectin concentration were shown in a study by Hua et al. [54]. These phases are the three classical domains observed for any polymer: dilute, semi-dilute, and concentrated [83]. In the first phase, the concentration of pectin ranged from 0 to 1.0% and it was possible that intermolecular distances were too large for the interaction of pectin molecules [54]. Thus, viscosity did not significantly change. At the range of pectin concentration from 1.0 to 2.5% (the secondary phase) the distances between pectin molecules were smaller, therefore a stronger interaction could occur. A substantial increase in viscosity was noted in this phase. In the third phase, at pectin concentration of 2.5 to 3.5% the aggregation of the chains of pectin leading to network formation was possible. Thus, viscosity increased significantly. 

The intrinsic viscosity is defined as the limiting value of the reduced viscosity at zero concentration of polymer [82]. This parameter is related to the ability of the polymer to increase the viscosity of its solution [84]. Values of intrinsic viscosity obtained by Kar and Arslan for pectins extracted from orange peel by EDTA, ammonium oxalate, and HCl were 0.309, 0.281, and 0.262 m^3^/kg, respectively [82]. The magnitude of this parameter for pectins extracted from sunflower head by using ammonium oxalate was 1.654 m^3^/kg [54]. The commercial high-methoxy citrus pectin had an intrinsic viscosity of 0.222 m^3^/kg [85]. 

Gel formation depends on the structure of pectins as well as on other factors, such as pectin and sugar concentrations, the presence of crosslinking agents, temperature, and pH. The structure of pectins is related to the degrees of methylation and acetylation (the percentage of galacturonic acid residues containing one acetyl group), the degree of amidation, molecular weight, and the heterogeneity of polymer chains [86]. During the course of the gelation process, three-dimensional networks holding water and solute molecules are created [87]. 

The mechanism of gel formation is different for high-methoxy (HM) pectins and low-methoxy (LM) pectins. HM pectins form a gel at pH < 3.5 and at high sugar concentrations (>55%) [78,88]. In acidic media, the dissociation of carboxyl groups of galacturonic acid residues is reduced. Therefore, the electrostatic repulsive forces are lower between pectin chains. High sugar concentration has an influence on reducing the hydration of pectin molecules [77]. Hydrogen bonds and hydrophobic forces play a significant role in gel formation [87]. A large number of hydrogen bonds between pectin molecules stabilize the gel structure. The hydrophobic interactions of methoxyl groups, connected with the reduction in contact area of these groups with water molecules, and a decrease in the energy of the system, also have an influence on its stabilization [89]. 

Generally, LM pectin gels are formed in the presence of divalent cations such as Ca^2+^ [90]. The required pH value is in the range of 2 to 6 and sugar is not necessary for gel formation [78]. An increase in ionic strength determines the lower concentration of Ca^2+^, which is required for gelation [90]. The gelation process of LM pectin under alkaline conditions was studied by Yang et al. [91]. An increase in pH from 3.5 to 8.5 resulted in an increase of gel hardness (determined using a texture analyser), which was connected with an increase in the degree of dissociation of galacturonic acid residues and next the de-esterification of pectin. However, a decrease in this parameter was observed at pH = 9.5, this was caused by a decrease in the molecular weight as a result of the β-elimination reaction. A similar dependence was observed for gel strength as determined on the basis of the storage modulus values. The gel point, was determined based on the dependence of storage and loss moduli on temperature, it was ~70 °C at pH = 3.5 and ~78 °C at pH = 9.5. Moreover, the impact of pH on gel formation was evaluated using scanning electron microscopy. Network structure was observed over the whole pH range (3.5–9.5) studied, however, the most compact structure was noted at pH = 8.5 [91]. 

**Figure 3:**
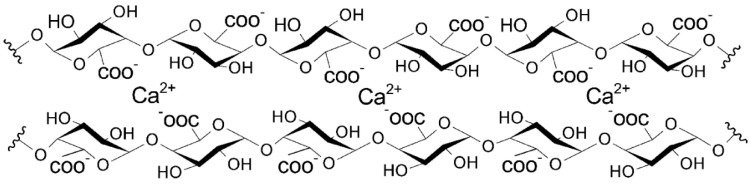
Scheme of “egg-box” model, based on: [77,98,100].

The mechanism of LM pectin gelation is related to the “egg-box” model describing the binding of Ca^2+^ by alginates [92,93]. Due to the similar structures and crosslinking behavior of alginates and pectins, the “egg-box” model was used to describe pectin and calcium ion interactions [94,95]. The differences in the binding processes of these polysaccharides (in chain–chain association—a pronounced shift of one galacturonate chain in relation to the other chain) were underlined by Braccini and Perez [96]. In the “egg-box” model, junction zones are formed by binding between Ca^2+^ and non-esterified galacturonic acid units [97]. X-ray and EXAFS studies have indicated that the interaction of calcium ions with polygalacturonate chains may occur via oxygen atoms in the carboxylate group, in the ring, in the glycosidic bond and in the hydroxyl group of the next residue [98]. Additionally, water molecules may interact with the calcium ions. A scheme of this model is presented in Figure 3. Gel formation at low pH (pH = 3) may be connected not only with the binding of calcium ions to pectin but also with hydrophobic interactions and the formation of hydrogen bonds [99]. In this case, the interaction of Ca^2+^ with other oxygen atoms in galacturonic acid residues was suggested.

Amidated LM pectins also form a gel in the presence of calcium ions [17]. In the gelation process of these pectins, fewer calcium ions are required than during gel formation by LM pectins. Amidated LM pectins are also more resistant to high calcium ion concentration. On the basis of conductometric measurements, the blockwise distribution of amide groups was suggested [101]. These authors implied that hydrogen bonds also take part in the gelation process of amidated LM pectins.

The degree and pattern of methylation have an influence on the number, stability, and cooperativeness of junction zones [97]. This is important for the mechanical and rheological properties of the gel. The number of non-esterified galacturonic acid units necessary to form stable junction zones varies from 6 to 20 [102]. Morris et al. proposed two models describing the long-range structure in gels [103]. The first model presents a pseudo rubber-like network in which crosslinking is extended and it does not have a point character (entropic gel). The second model is connected with the formation of branched fibrous structures (enthalpic gel).

Ngouémazong et al. studied the influence of de-methylesterification on the characteristics of pectin-Ca^2+^ gels [97]. Pectins with a different degree of methylation (DM) and an absolute degree of blockiness (DB_abs_) were investigated. DB_abs_ is related to the amount of non-methylesterified galacturonic acid residues released as result of endo-polygalacturonase action on pectin relative to the total amount of galacturonic acid. The impact of Ca^2+^ concentration on the nature of the gels was also studied. Pectins with very low values of DM and high values of DB_abs_ (DM = 10.1% and 14.9%, DB_abs_ = 64.4% and 79.4%, respectively) formed strong gels at different Ca^2+^ concentrations. Generally, the decrease in DM causes an increase in the gelation ability of LM pectins [90]. The ability to form a gel, expressed as gel strength, was determined on the basis of the values of the storage modulus (G’). For these systems, the values of G’ were in the range of 3000–64,000 Pa. The occurrence of long blocks of non-esterified galacturonic acid units has an influence on the formation of stable and strong junction zones. The strength of pectin-Ca^2+^ gels was also studied by Kim and Wicker [104]. High-methoxy pectins were de-esterified using two pectin methylesterases from Valencia orange: U-PME and B-PME differ in peptide composition and cationic character. Pectins modified by U-PME formed gels with higher values of G’ (G’ = 502.5 Pa at 0.4 Hz) than those formed by B-PME (G’ = 218.7 Pa at 0.4 Hz) at a CaCl_2_ concentration of 35 mM. These pectins had a similar DM: 61% and 63%, respectively. The differences in gelling properties were connected with the different distributions of charged carboxyl groups in these pectins. 

Gilsenan et al. investigated the gelation process of LM pectins in acidic media without the addition of Ca^2+^ (or a cosolute) [105]. They suggested that gel formation is connected with the dimerization of antiparallel polymer chains forming three-fold helices. This association is significant at very low pH values on account of uncharged carboxyl groups. In acidic media the protonation of carboxyl groups occurs. Electrostatic repulsion is reduced as a result of protonation. Moreover, uncharged carboxyl groups may form hydrogen bonds inside the polymer molecule and between two neighboring chains [106].

Jarvis and Apperley suggested that calcium pectate transitions from a two-fold to a three-fold conformation upon drying [107]. This is related to the polymorphic phase transition of calcium pectate. Calcium alginate, which was originally described by the “egg-box” model, retains two-fold conformation [94].

## 4. Crosslinking Agents

Apart from calcium, other divalent and trivalent cations can also bind to pectin chains [102]. The influence of the addition of Ca^2+^, Mg^2+^, and Fe^2+^ on the rheological properties of the modified cell wall polysaccharide matrix (MPS) obtained from apple and containing large amounts of pectin was investigated by Mierczyńska et al. [9]. The presence of ions such as Ca^2+^ and Fe^2+^ caused an increase in the viscosity of MPS solutions. Furthermore, an increase in the viscosity was observed with the increasing concentration of these ions. In the case of the addition of Mg^2+^ to MPS solutions, a decline in viscosity with the increasing concentration of these ions in solution was observed. The higher values in the viscosity of MPS solutions in the presence of calcium ions were connected with the crosslinking of low-methoxy pectins with these ions according to the “egg-box” model [4,78,103,108]. It is likely that the increase in the viscosity of MPS solutions with the addition of divalent iron ions may be caused by the interaction of pectins with Fe^2+^, which is similar to the interactions, which occur between pectins and Ca^2+^. The binding of Fe^2+^ to citrus pectins was also recently investigated by Celus et al. [109]. On the basis of adsorption isotherms, the authors noted that the structural properties of these pectins such as DM and DB_abs_ had an influence on iron ions binding capacity. DM mainly determined the maximum adsorption capacity while DB_abs_ mostly influenced the pectin-iron ions interaction energy. They suggested that chemically de-methylesterified pectins with DM in the range of 20–67% and DB_abs_ in the range of 5–44%, may interact with iron ions according to the “egg-box” model. Debon and Tester proposed that Fe^3+^ may interact with the carboxyl groups of the galacturonic acid residue in acidic conditions [110]. 

The interactions between Ca^2+^, Ba^2+^, Mg^2+^, and Zn^2+^ and polygalacturonate were studied using measurements of viscosity and turbidity, isothermal titration calorimetry and molecular dynamics simulations [111]. The weak binding of Mg^2+^ to polygalacturonate was observed and this was explained by the high affinity of this ion for water, whereas Ba^2+^, Ca^2+^, and Zn^2+^ interacted with the polygalacturonate to form network. The binding mode of Ba^2+^ and Ca^2+^ to carboxylate ions was bidentate (two oxygen atoms of the carboxylate ion are involved in this process), while Zn^2+^ was bound in monodentate coordination (by one oxygen atom of the carboxylate ion). A two-step binding mechanism of Ba^2+^, Ca^2+^, and Zn^2+^ with polygalacturonate was proposed, where in the first step monocomplexes and point-like cross-links are formed while the second step is connected with the formation of dimers [111]. Fang et al. have proposed a similar binding mechanism of Ca^2+^ to LM pectins [95]. In the case of Mg^2+^-polygalacturonate interactions, polycondensation was suggested when specific conditions are provided [111]. 

Wellner et al. investigated the interactions between different divalent cations and potassium pectate [98]. A similarity in band shifts in the FT-IR spectra for ions such as Ca^2+^, Sr^2+^, Zn^2+^, Ni^2+^,Cu^2+^, Pb^2+^, and Cd^2+^ was observed. The band shifts were found in the 1200–950 cm^−1^ region, especially the shift of the 1018 cm^−1^ band (the main maximum was connected with C–C and C–O vibrations of the pyranoid ring). These results suggest that these cations were linked with the pectate chain in a similar way. In the case of the Mg^2+^ band at 1004 cm^−1^, it was observed in pectate solution, but this band had a lower intensity than the bands of other cations studied.

The binding ability of Ca^2+^ and Zn^2+^ by low-methoxy pectins was investigated by equilibrium dialysis in acidic and neutral media by Debon and Tester [110]. In strong acidic solutions (at pH = 1) these ions were not bound by pectins due to the presence of protonated carboxyl groups which prevented electrostatic interactions. In neutral solutions (in water) the binding of calcium and zinc ions by pectins was evident as a consequence of electrostatic interactions between charged carboxyl groups of pectins and these ions. The binding process of Ca^2+^ and Zn^2+^ by pectins was also studied by Schlemmer [112]. The highest degree of binding was noticed at low ionic strength and pH from 5.0 to 7.5 in diluted solutions of hydrochloric acid/sodium hydroxide. In the same pH range, at higher ionic strength and in the bicarbonate and carbon dioxide buffering system, pectin did not bind or only bound to a limited extent with these ions.

Further studies of metal ions—pectin systems are still required to determine the interaction mechanism. It is probably that the binding of divalent and trivalent metal ions by pectin is similar to the above-described “egg-box” model. However, the differences in the atomic properties of elements may have an influence on their binding mode to pectin. The application of various metal ions in the gelation process of LM pectins may modify the structure and properties of gels and therefore food products. The addition of some elements to food may also have a positive effect on the human body.

## 5. Interactions of Pectins with Other Natural Compounds

### 5.1. Cellulose

The model of the primary cell wall, called the ‘tethered network model’, describes the interaction between cell wall polysaccharides [113]. According to this model, xyloglucans coat cellulose microfibrils and tether them while pectins form an independent network in which this cellulose/xyloglucan network is embedded. However, there are suggestions connected with the interactions of cellulose with pectin [114,115].

Pectins extracted from sugar beet and potato cell wall materials (AIR), commercial branched arabinans, debranched arabinans, galactan, and commercial citrus pectins were investigated for their binding capacity to Avicel microcrystalline cellulose in a study by Zykwinska et al. [116]. Moreover, the binding ability of pectins isolated from sugar beet AIR to the primary cell wall (PCW) cellulose from the same material was studied. The amount of adsorbed polysaccharide was determined on the basis of the difference between the uronic acid or total neutral sugar content in the polysaccharide samples and polysaccharide/cellulose samples. Pectins isolated from sugar beet and potato AIR had the ability to bind to Avicel cellulose (in vitro) presumably through side chains of arabinans and galactans and the complexes obtained had a low level of reversibility. The authors suggested that hydrogen bonds could be formed [116]. A model of pectins associated with cellulose through galactan side chains in apple cell walls was also proposed by Oechslin et al. on the basis of results from the enzymatic degradation of cellulosic residue extracted from apples using cellulase and enzymes degrading pectic substances [117]. Changes in the rheological properties of sugar beet microfibrillated cellulose suspension as a result of the addition of apple HM pectins were observed by Agoda-Tandjawa et al. [118]. The values of the G’ and G’’ moduli were higher for the apple HM pectin/cellulose system (without the addition of NaCl) than for the cellulose suspension (pectin/cellulose proportion: 0.5/0.5) which indicates the enhancement of the viscoelastic properties of the cellulose suspension. It is likely that these pectins bind to cellulose through side chains.

The binding ability of branched arabinans and galactans was similar to that of pectins extracted from sugar beet and potato AIR [116]. In the case of debranched arabinans, the adsorption of cellulose was higher than for other polysaccharides. Therefore, it has been assumed that the branching may reduce the binding process of pectin and cellulose. In the case of the binding of pectins isolated from sugar beet AIR to the PCW cellulose, the amount of adsorbed pectin was higher for the PCW cellulose than for the Avicel cellulose. This may be related to the different crystallinity index and surface availability of these studied celluloses [116,119].

On the basis of the investigation of the adsorption of commercial citrus pectins to cellulose it was observed that these pectins did not bind to cellulose [116]. This may be due to the low content of side chains in these pectins extracted in strongly acidic conditions. In a study by Agoda-Tandjawa et al. the addition of citrus HM pectins to cellulose did not change the rheological properties of cellulose [118].

**Figure 4:**
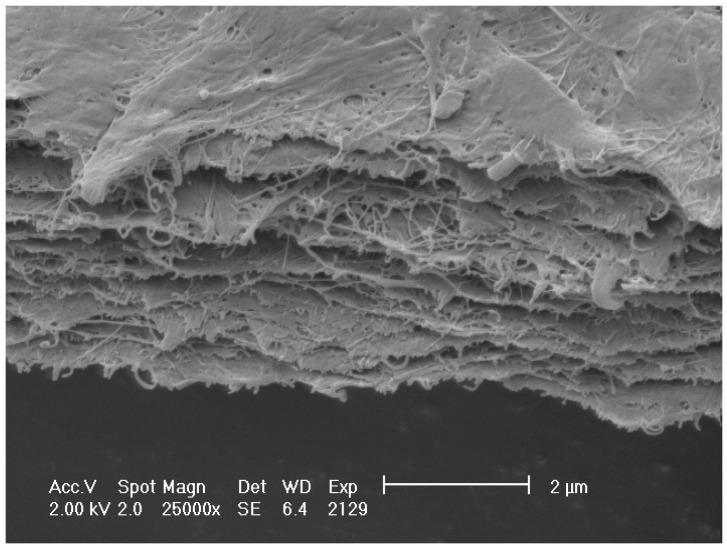
SEM micrograph of bacterial cellulose/pectin composite.

SEM micrograph of bacterial cellulose/pectin composite is presented in Figure 4.

### 5.2. Hemicellulose

Covalent linkage between pectin and hemicellulose—xyloglucan was proposed in the model of primary plant cell walls by Keegstra et al. [120]. They suggested a covalent link between these two polysaccharides through side chains of rhamnogalacturonan I—arabinogalactans and reducing ends of xyloglucan. The presence of xylose and uronic acid units in oligomeric fragments, obtained as the result of enzymatic digestion of sycamore cell walls, was the evidence for the presence of linkages between xyloglucan and pectin. Xylose and uronic acid units were assumed to be only part of the structure of xyloglucan and pectin, respectively. At a later date, the presence of uronic acid residues in hemicelluloses and also xylose and 2-*O*-methyl-xylose in pectin was reported [26,121]. However, further evidence for pectin-xyloglucan linkages was provided. Thompson and Fry showed that some part of xyloglucan was covalently linked to pectins, probably through side chains—arabinogalactans, in cell walls of cell-suspension cultures of rose [122]. The formation of linkages between pectins and xyloglucans was observed in in-vitro biosynthesis in the Golgi apparatus by Abdel-Massih et al. [123]. However, this complex may not be the same as complexes discovered in some cell walls. The complex synthesized in the Golgi apparatus was not linked with cellulose whereas linkages of pectin with xyloglucan forming hydrogen bonds with cellulose were proposed [120,123]. The structure of this first complex was based on a backbone of xyloglucan with attached pectin in contrast to the model proposed by Keegstra et al. Covalent linkages between pectin and xyloglucan were also found in cell-suspension cultures of different angiosperm plants such as rose, sycamore, Arabidopsis, spinach, tomato, barley and maize [124].

Tan et al. indicated covalent linkages between pectin, arabinoxylan, and arabinogalactan protein (AGP) in the cell wall of *Arabidopsis*. In this structure, named APAP1, arabinoxylan is linked to rhamnogalacturonan I or arabinogalactan of the AGP while rhamnogalacturonan I with attached homogalacturonan is linked to the arabinogalactan domain of the AGP [125]. Therefore, some arabinogalactan proteins may play a role in the formation of a network between polysaccharides and structural proteins of cell walls. This model differs from the one described in a study by Keegstra et al. and the ‘tethered network model’.

### 5.3. Ferulic Acid

In the sugar beet cell wall ferulic acid residues ester-linked to the arabinan and galactan side chains of pectins were found [126]. This connection may occur through O-2 of the α-1,5-arabinose units of the arabinan chain and O-6 of the β-1,4-galactose units of the galactan chain [127]. It was suggested that small amounts of ferulic acid residues were attached to O-5 of the α-1,5-arabinose units of the arabinan chain [128]. Ferulic acid esters may form dehydrodimers as a result of an oxidative reaction in the cell wall and through the treatment of pectins isolated from sugar beet with oxidases or one-electron oxidants [126,129]. In plant cell walls, 4 dimer forms were found: 5-5′, 8-O-4′, 8-5′, and 8-8′ dehydrodiferulates [126]. The percentage of dimers in the total ferulic acid in sugar beet root was approximately 20% [130]. In these pectins, the main dimer forms were 8-5′ and 8-O-4′ ferulate dehydrodimers. In a study by Oosterveld et al. after the application of hydrogen peroxide and peroxidase to the oxidative crosslinking of sugar beet pectins, a decrease in the amount of ferulic acid monomers and an increase in the amount of dehydrodiferulates were observed [129]. The greatest increase in the sample was noted in the amounts of 8-5′ and 8-O-4′ ferulate dehydrodimers.

Ralet et al. isolated fractions containing dehydrodiferulates using acid and enzymatic hydrolysis of sugar beet AIR and hydrophobic interaction chromatography [126]. Dehydrodiferulates attached to O-2 or O-5 of non-reducing arabinose units were found. One isolated compound consisted of a dimer of arabinose, a dimer of galactose and dehydrodiferulate linked to O-2 of a non-reducing arabinose unit and O-6 of a non-reducing galactose unit. On the basis of these studies, it has been suggested that arabinose and galactose units of pectin side chains are covalently crosslinked by diferulic bridges in the sugar beet cell wall.

Ferulic acid has an influence on emulsifying ability and stability, and the surface activity of sugar beet pectins [131]. It may reduce interfacial tension by adsorption at the oil/water interface. The availability of ferulic acid groups is important for the emulsification properties of sugar beet pectin [132].

### 5.4. Protein

Proteinaceous materials covalently bound to the side chains of pectins (mainly to galactose and arabinose units) had an impact on the emulsifying properties of sugar beet pectins [131]. Funami et al. showed that the decrease in the amount of protein in the sugar beet pectin, as a result of enzymatic modification (using pepsin or acidic proteinase), caused an increase in the average droplet diameter in O/W emulsions, creaming of the oil/water emulsions during incubation at 60 °C and a decrease in the amount of pectins adsorbed at the oil/water interface [133]. This indicates that the ability to emulsify sugar beet pectin was reduced after enzymatic modification. 

Proteins may adsorb at the oil/water interface and lower the interfacial tension. Thus, they may prevent the aggregation of droplets and stabilize an emulsion [134]. The influence of protein content on droplet size in oil/water emulsions was also observed in a study by Chen et al. [135]. A significant decrease in droplet size was found due to an increase in protein content from 0.5 to 3.0%. At values above 3.0%, protein a substantial change in droplet size was not observed. The presence of significant amounts of side chains of sugar beet pectins had an impact on the adsorption of these pectins at the oil/water interface. These side chains caused steric hindrance limiting the number of adsorbed pectin molecules. Thus, large amounts of proteins associated with pectin chains may not cause a further decrease in droplet size of the oil/water emulsion. 

The emulsifying properties of sugar beet pectins depend on their chemical structure (monosaccharide composition, degrees of methylation and acetylation) and the presence of attached proteins and ferulic acid residues [132,133,135]. The investigation of the presence of protein and ferulic acid residues bound to pectin extracted from different sources has a potential significance in designing food emulsions. 

Protein may also be added to pectins for the modification of their physicochemical properties. Tamnak et al. studied the influence of mixing and the process of the conjugation of high-methoxy citrus pectin and pea protein isolate on the physicochemical properties of these systems [136]. Protein-polysaccharide conjugates may be formed as a product of the Maillard reaction in which the carbonyl group of a reducing sugar residue of polysaccharide reacts with an amino group of protein [137]. The rate of a Maillard reaction depends on the protein/polysaccharide ratio, the nature of each reagent, pH, temperature, and the relative humidity [138]. Tamnak et al. observed that the ratio of pectins to proteins had a bigger influence on the solubility of pectin-protein conjugates than the time of incubation [136]. The solubility of these systems increased in comparison with the solubility of pea protein isolate and decreased in comparison with the solubility of pectin. After a longer incubation time a slight decline in the solubility of the hybrid polymer was noted. This may be a consequence of the amphiphilic character and high molecular weight of the conjugates [139]. The proportion of pectins to proteins had a significant impact on the moisture content of the conjugates [136]. With the increase in the ratio of pectin to protein, rising from 1/1 to 3/1, an increase in the moisture content was observed. This may be related to a higher water-binding capacity by pectins in the conjugation process. The presence of pectin/protein conjugates caused a smaller droplet size and a greater stability of the oil/water emulsion than the presence of pectin alone or mixtures of pectin and protein in the oil/water emulsion. This may be related to the higher level of adsorption of conjugates at the oil/water interface due to their amphiphilic character. Thus, the conjugates may have better emulsification properties than pectin or protein alone. The highest level of stability and the smallest droplet size were observed for emulsions stabilized by a pectin/protein conjugate (ratio of 3/1). An excess of protein may have an influence on the increase in pH and a reduction in the stability of the emulsion. According to Turgeon and Laneuville, a greater proportion of protein to polysaccharide may cause the flocculation of droplets in emulsions [140]. 

High methoxy pectins stabilize acid milk products [78]. At an acidic pH (the typical pH of yoghurt is 4) caseins have a poorly positive net charge. Carboxyl groups of pectins are dissociated at this pH and may react with casein particles. In this way, pectins prevent the coagulation of proteins by steric stabilization [141]. The influence of the addition of high methoxy pectins on the stability of acid milk drinks was also investigated by Tromp et al. [142]. On the basis of studies of the degree of pectin adsorption, pectin concentration in serum (continuous) phase of colloidal system and using techniques of confocal scanning laser microscopy and fluorescence recovery after photobleaching it was concluded that only a small amount of pectins are adsorbed on casein micelles, the remaining part (up to 90%) of pectins fill spaces in the network which is formed by complexes of pectin and casein micelles. The formation of this network has an influence on the stability of acid milk drinks. The removal of non-adsorbed pectin from these products did not cause a reduction in stability, however the presence of pectin, not interacting directly with casein, is essential in the production process of acid milk drinks.

The interaction of pectin with protein is important for the food industry. The emulsification properties of pectin may be improved by the addition of protein and also pectin may stabilize systems containing protein, such as acid milk products. 

### 5.5. Starch 

On the basis of rheological measurements, higher values of shear stress, as a function of shear rate, were found for the high-methoxy pectin/sucrose/modified potato starch systems than for starch solution [143]. This may be related to the binding of pectins to starch or a higher co-solute concentration in the mixture. An increase in pseudoplasticity was observed in systems containing acetylated distarch phosphate at a concentration of 4.5% *w*/*w* and acetylated distarch adipate at a concentration of 3.5% and 4.5% *w*/*w* in comparison with starch alone. A decrease in pseudoplasticity was noted in the case of the acetylated distarch phosphate system at a modified starch concentration of 2.5% and 3.5% *w*/*w*. Higher values of G’ and G”, and lower values of loss tangent (tan δ) with an increase in low-methoxy pectins concentration were observed in low-methoxy pectin/native tapioca starch systems with calcium ions [144]. This was the result of an increase in the viscoelasticity of the system. This, in turn, was caused by starch molecules enhancing the pectin network through ionic interactions [145]. 

In the case of a high-methoxy pectin/sucrose/starch system, higher pasting temperatures and higher values of peak and final viscosities were observed than for the starch solution [143]. This may be connected with the formation of hydrogen bonds between sucrose and starch. The pasting temperature is the temperature above which gelatinization occurs in aqueous starch solution [146]. The addition of low-methoxy pectins (with Ca^2+^) to native tapioca starch solution did not cause a change in the pasting temperature [144]. However, an increase in this parameter was observed in pectin/inulin/potato starch systems in comparison to a starch solution used in the study by Witczak et al. [146]. Pectins and inulin may reduce the process of the binding of water molecules to starch amorphous regions and, in this way, they may increase the pasting temperature. In contrast to results obtained by Gałkowska et al. [143], these polysaccharides caused a decrease in the peak and final viscosities in comparison with the starch solution [146]. Agudelo et al. observed an increase in the cold paste viscosity, hot paste viscosity, and peak viscosity as a result of the addition of low-methoxy pectins (with Ca^2+^) to starch solution [144].

Pectins and sucrose also had an influence on the textural properties of high-methoxy pectin/sucrose/starch systems [143]. The values of back-extrusion parameters (textural parameters obtained as a result of the back-extrusion test) were higher for mixtures than for starch solutions. It is likely that pectins and sucrose could reinforce the starch matrix. Increases in the values of extrusion parameters were also observed in the low-methoxy pectin/native tapioca starch system with the addition of calcium ions [144].

Pectin/starch systems may be a viable alternative to modified starch commonly used in the food industry. During preparation of fruit fillings and pastries high temperatures cause the degradation of native starch while repeated freezing and thawing of products during distribution change its rheological properties and contribute to syneresis [145]. Low-methoxy pectins added to native tapioca starch counteracted syneresis and maintained stability at high temperatures. Moreover, the pectin/starch system had similar rheological properties to modified starch.

### 5.6. Chitosan

Pectins may interact with chitosan to form a polyelectrolyte complex (PEC) [147]. Due to the presence of their functional groups, the positively charged amino groups of chitosan and the negatively charged carboxyl groups of pectin, electrostatic attractions occur between these polysaccharides [148]. In addition, interactions such as hydrophobic forces, van der Waals interactions, hydrogen bonds, and coordinate bonds are possible between these polymers in their complexes. Different factors have an influence on the stability of these systems, for example, ionic strength, charge density, pH, and temperature [149]. 

PEC films are formed mainly in a pH range between the pK_a_ ranges of chitosan and pectin [147]. The pK_a_ of pectin varies from 3.5 to 4.5 while the pK_a_ of chitosan varies from 6.2 to 7.0 [150,151]. On the basis of zeta potential measurements, the maximum yield of PEC formation (60.2%) was determined based on the dry weight of pectin (DE = 72.0%), chitosan (DA = 9.1%), and a complex was obtained at pH 5.5 with a pectin/chitosan ratio of 4.3/1.0 [147]. This could be connected with the presence of more than one half of the ionized groups of pectin and chitosan. At pH 5.0 and 5.5 an increase in the homogeneity of PEC was noticed. In a study by Coimbra et al. (2011) PEC films obtained from chitosan (DA ∼ 28%) and pectin (DE ∼ 42%) with a pectin/chitosan ratio of 2/1 and 1/1 had an almost identical percentage composition (30.4% and 31.9% of chitosan, respectively) independent of the different proportions of these polysaccharides [152]. The value of DE was higher than the value of DA; therefore, a lower number of ionizable groups of pectin than chitosan are present per mass unit. Thus, a greater amount of pectin than chitosan is required in the interaction between pectin and chitosan in a 1:1 stoichiometry (for similar dissociation degrees). The dissociation degree of polysaccharide groups, which could be ionized, depends on the pH of the solution. Therefore, the pH of the solution has an influence over the stoichiometry of the reaction. In this study the value of the pH was the same (pH = 4.5), so the stoichiometry did not change with different ratios of these polysaccharides. Thus, the PEC films were characterized by a similar percentage composition of pectin and chitosan. Additionally, on the basis of thermogravimetric analysis, it was observed that PEC films were degraded at a lower temperature than chitosan [147].

Chitosan/pectin systems alone and those with the addition of calcium ions or *N*-hydroxysuccinimide (NHS) and 1-ethyl-3-(3-dimethylaminopropyl) carbodiimide (EDC) were studied by Chen et al. [153]. Calcium ions were used as the crosslinking agents of pectins, whereas NHS and EDC enabled the formation of covalent bonds between pectin and chitosan. The tensile strength of membranes increased with a higher weight ratio of pectin to chitosan. The highest value of this parameter was for the chitosan/pectin ratio of 50/50. For this system the carboxyl groups/amino groups ratio was greater than for other systems of this type, thus interactions between pectin and chitosan were stronger and the tensile strength was higher. The addition of calcium ions or NHS and EDC to chitosan/pectin systems caused an increase in the tensile strength of the membranes. The hydrophilicity of the chitosan/pectin films was determined on the basis of measurements of contact angles. The smallest contact angle was produced by a chitosan/pectin system with a ratio of 50/50 (among systems without additional reagents). Therefore, hydrophilicity reached its highest level for this system. The presence of calcium ions in the system had an influence on a decrease in the value of the contact angle, whereas the addition of NHS and EDC did not cause any substantial change in this parameter. Increasing the ratio of pectin to chitosan caused an increase in the water uptake capability of chitosan/pectin systems. However, a decline in the water uptake ability was observed for systems containing additional calcium ions or NHS and EDC, this may be connected to a decrease in the intermolecular space as a result of the formation of covalent bonds which are stronger than ionic bonds [154] and with stronger interactions between pectin molecules as a result of the presence of calcium ions [153]. 

In a study by Maciel et al. the FT-IR spectrum of chitosan showed an asymmetric stretching vibration arising from C=O in the amide group of the acetylated chitosan residues (the amide I band) at 1643 cm^−1^ and an overlapping band at 1600 cm^−1^ arising from the bending vibration of N–H and the stretching vibration of C–N of the amide group (the amide II band) and the bending vibration of N–H of the amino group [147]. In the spectrum of pectin the asymmetric stretching vibration of C=O in carboxylate groups occurred at 1631 cm^−1^. On the basis of an analysis of the PEC spectrum (pectin/chitosan ratio of 4.3/1.0) a broad band at 1600–1500 cm^−1^ was found which pointed to interactions between chitosan and pectin. The FT-IR spectra of PEC films received by Coimbra et al. showed a similarly broad band at 1660–1500 cm^−1^ [152]. Chen et al. also observed a peak at 1598 cm^−1^ in the chitosan/pectin system (chitosan/pectin ratio was 70/30) and at 1589 cm^−1^ in this system with the addition of calcium ions [153]. In chitosan/pectin membranes containing NHS and EDC a band at 1557 cm^−1^ arising from the amide bond between carboxyl groups and amino groups was found. It is possible that at 1625–1560 cm^−1^ and 1550–1505 cm^−1^ two new bands were present that were connected with the asymmetric and symmetric bending vibrations of the protonated amino group [152]. These new bands may overlap with the other band found previously.

The pectin/chitosan complex may be used to entrap biologically active compounds such as anthocyanins [147]. These systems changed color depending on pH, which was connected with changes to the chemical structure of anthocyanin molecules. It has potential applications in the control of pH changes in food products associated with food spoilage.

A summary of the binding mechanism and properties of complexes is shown in Table 2.

## 6. Conclusions

This review demonstrated that pectins are useful for the food industry due to their ability to gel and link with various components under a wide range of conditions. Hydrogen bonds and hydrophobic interactions take part in the gel formation of high-methoxy pectins. In turn, the low-methoxy pectin gels form at pH = 2–6 and in the presence of crosslinking agents such as calcium ions according to the “egg-box” model. The interaction between pectins and other divalent metal ions is also possible. The substitution of calcium ions by other divalent cations, for example, zinc or iron ions in the gelation process of LM pectin could have an influence on the pro-health values of food products delivering important elements for the human body. Pectins can also effectively bind with other natural components, such as cellulose through pectin side chains, interact with starch, form polyelectrolyte complexes with chitosan, and take part in Maillard reactions with protein. Both pectin and starch are commonly used in the food industry, therefore the application of pectin/starch systems with the addition of sucrose may be used to improve the textural properties of food products. The combination of pectin, chitosan, and anthocyanin may be used to monitor the quality of food products. Properly prepared pectin/protein systems may be applied to the production of food emulsions due to their emulsification properties.

Pectins, as natural biocomponents, should be the focus of both the food industry and the bioeconomy, since they are also extracted from cell walls just as cellulose and hemicellulose are. However, due to the complexity of the pectin family and the dynamic structural changes during plant organ development, a more intensive study on their structure-related properties is necessary. Fractionating using different solvents at well-defined development stages and in-depth studies of the molecular structure and properties within each fraction and stage is one possible way to proceed.

## Figures and Tables

**Table 1:** Galacturonic acid content and yield of pectin depending on the source and method of extraction.

Pectin Source	Galacturonic Acid Content (%)	Yield (%)	Extraction Method	References
Apple pomace	~21–44	~10–17	subcritical water (t = 5 min, T = 130–170 °C, solid/liquid ratio 1:30)	[40]
Black currant	37.1	-	citric acid (t = 30 min, T = 90 °C, pH = 2.5, solid/liquid ratio 1:50)	[41]
Black mulberry pomace	~29–43	~9–14	hydrochloric acid, microwave-assisted extraction (irradiation time 10–30 min, pH = 2, power 300–900 W, solid/liquid ratio between 1:15 and 1:30)	[42]
Cacao pod husks	~60 (of total sugar content)	~11	nitric acid (t = 30 min, T = 100 °C, pH = 3.5)	[43]
Carrot	16.5	-	citric acid (t = 30 min, T = 90 °C, pH = 2.5, solid/liquid ratio 1:50)	[41]
Gold kiwifruit pomace	~82–85 (of total non-starch polysaccharides)	~4	citric acid (t = 1 h, T = 50 °C, pH = 2.2, pomace/acid solution ratio 1:3 *w*/*v*), water (t = 30 min, T = 5 °C, pomace/water ratio 1:3 *w*/*v*) and enzymatic extraction (t = 30 min, T = 25 °C, enzyme: Celluclast 1.5 L)	[44]
Mango peel	~29–35 (T = 20 °C) ~52–53 (T = 80 °C)	~2 ~17	citric acid, conventional extraction (t = 2 h, pH = 2.5, solid/liquid ratio 1:40) and ultrasound-assisted extraction (t = 15 min, pH = 2.5, solid/liquid ratio 1:40)	[45]
Okra (*Abelmoschus esculentus* L.)	~43–63	11–15	aqueous extraction, phosphate buffer (t = 1 h, T = 80 °C, pH = 6.0, solid/liquid ratio 1:15)	[46]
*Opuntia ficus indica* cladodes	~69	~19	acidified water, ultrasound-assisted extraction (t = 70 min, T = 70 °C, pH = 1.5, solid/liquid ratio 1:30)	[47]
Orange juice wastes	~46–74	~1–11	hydrochloric acid, ohmic extraction (T = up to 90 °C, pH = 1.5–4, voltage gradient 5–30 V/cm, solid/liquid ratio between 1:10 and 1:40)	[48]
Orange peel	~66–70	~14–18	hydrochloric acid (t = 1 h, T = 80–82 °C, pH = 1.5, solid/liquid ratio 1:50), microwave heating (t = 5–15 min, power 0.45–0.9 kW)	[49]
Peach	26	-	citric acid (t = 30 min, T = 90 °C, pH = 2.5, solid/liquid ratio 1:50)	[41]
Pistachio green hull	~65	~22	citric acid (t = 30 min, T = 90 °C, pH = 0.5, solid/liquid ratio 1:50)	[50]
Plum	23.8	-	citric acid (t = 30 min, T = 90 °C, pH = 2.5, solid/liquid ratio 1:50)	[41]
Pomegranate peel	~70–82	~3–9	citric acid (t = 40–150 min, T = 70–90 °C, pH = 2–4)	[51]
Raspberry	23.1	-	citric acid (t = 30 min, T = 90 °C, pH = 2.5, solid/liquid ratio 1:50)	[41]
Soy hull	~67–69	~16–21	hydrochloric acid (t = 1 h, T = 90 °C, solid/liquid ratio 1:10)	[52]
Strawberry	33.9	-	citric acid (t = 30 min, T = 90 °C, pH = 2.5, solid/liquid ratio 1:50)	[41]
Sugar beet pulp	~54–64	~1–9	hydrochloric acid (t = 1 h, T = 80 °C, pH = 1.5, solid/liquid ratio 1:20)	[53]
Sunflower head	86	-	ammonium oxalate (t = 45 min, T = 85 °C)	[54]
	~50 of total sugar content	~9	citric acid, ultrasound-assisted extraction (t = 32 min, pH = 3.2, solid/liquid ratio 1:15)	[55]
Yellow passion fruit peel	~48–72	~4–8	nitric acid, moderate electric field extraction (t = 5–60 min, pH = 1–5, voltage 30–100 V, solid/liquid ratio 1:30)	[56]

**Table 2:** Interaction mechanism and the main properties of systems of pectin with different components.

Components	Interaction Mechanism	The Main Properties of the Complex	References
Avicel cellulose	By pectin side chains—arabinans and galactans; possible formation of hydrogen bonds	Low reversibility of complex	[116]
Sugar-beet microfibrillated cellulose	By pectin side chains	Enhancement of viscoelastic properties of cellulose suspension	[118]
Ferulic acid/protein	Covalently linked to pectin side chains, mainly to arabinose and galactose residues (in sugar beet cell wall)	Improvement in emulsifying ability and stability, surface activity of sugar beet pectins	[131]
Protein	Maillard reaction: carbonyl group of a reducing sugar residue of pectin reacting with an amino group of protein	Changes in solubility; amphiphilic character; high molecular weight; better emulsification properties	[136]
Starch	possible enhancement of pectin network through ionic interactions	Increase in the viscoelasticity, values of starch pasting parameters and extrusion parameters	[144,145]
Chitosan	Formation of a polyelectrolyte complex: electrostatic interaction between oppositely charged groups (pectin: COO^−^, chitosan: NH_3_^+^); other possible interactions: hydrogen bonds, coordinate bonds, van der Waals interactions and hydrophobic forces	Homogeneous PEC films; degradation of PEC films at lower temperature than decomposition of chitosan (thermogravimetric analysis)	[147,148]
Chitosan + calcium ions or NHS/EDC	Calcium ions as crosslinking agents of pectins; NHS/EDC: formation of covalent bonds between pectin and chitosan	Higher tensile strength of membranes and lower water uptake ability	[153]
